# Single nucleotide polymorphism discovery in rainbow trout by deep sequencing of a reduced representation library

**DOI:** 10.1186/1471-2164-10-559

**Published:** 2009-11-25

**Authors:** Cecilia Castaño Sánchez, Timothy PL Smith, Ralph T Wiedmann, Roger L Vallejo, Mohamed Salem, Jianbo Yao, Caird E Rexroad

**Affiliations:** 1 West Virginia University, Animal and Nutritional Sciences, Morgantown, WV, 26506, USA; 2 USDA/ARS/NCCCWA, 11861 Leetown, Kearneysville, WV, 25430, USA; 3 USDA/ARS/USMARC, PO Box 166, Clay Center NE, 68933, USA

## Abstract

**Background:**

To enhance capabilities for genomic analyses in rainbow trout, such as genomic selection, a large suite of polymorphic markers that are amenable to high-throughput genotyping protocols must be identified. Expressed Sequence Tags (ESTs) have been used for single nucleotide polymorphism (SNP) discovery in salmonids. In those strategies, the salmonid semi-tetraploid genomes often led to assemblies of paralogous sequences and therefore resulted in a high rate of false positive SNP identification. Sequencing genomic DNA using primers identified from ESTs proved to be an effective but time consuming methodology of SNP identification in rainbow trout, therefore not suitable for high throughput SNP discovery. In this study, we employed a high-throughput strategy that used pyrosequencing technology to generate data from a reduced representation library constructed with genomic DNA pooled from 96 unrelated rainbow trout that represent the National Center for Cool and Cold Water Aquaculture (NCCCWA) broodstock population.

**Results:**

The reduced representation library consisted of 440 bp fragments resulting from complete digestion with the restriction enzyme *Hae*III; sequencing produced 2,000,000 reads providing an average 6 fold coverage of the estimated 150,000 unique genomic restriction fragments (300,000 fragment ends). Three independent data analyses identified 22,022 to 47,128 putative SNPs on 13,140 to 24,627 independent contigs. A set of 384 putative SNPs, randomly selected from the sets produced by the three analyses were genotyped on individual fish to determine the validation rate of putative SNPs among analyses, distinguish apparent SNPs that actually represent paralogous loci in the tetraploid genome, examine Mendelian segregation, and place the validated SNPs on the rainbow trout linkage map. Approximately 48% (183) of the putative SNPs were validated; 167 markers were successfully incorporated into the rainbow trout linkage map. In addition, 2% of the sequences from the validated markers were associated with rainbow trout transcripts.

**Conclusion:**

The use of reduced representation libraries and pyrosequencing technology proved to be an effective strategy for the discovery of a high number of putative SNPs in rainbow trout; however, modifications to the technique to decrease the false discovery rate resulting from the evolutionary recent genome duplication would be desirable.

## Background

Single Nucleotide Polymorphisms (SNPs) are highly abundant markers which are evenly distributed throughout the genome and can be functionally relevant[1]. They are suitable markers for fine mapping of genes and candidate gene association studies aimed at identifying alleles potentially affecting important traits. Technologies that enable simultaneous analysis of thousands of SNPs have permitted genome-wide association studies for complex traits in humans [2], chicken [3], cattle [4-6] and sheep [7]. Additionally, reduced representation libraries and pyrosequencing technologies have facilitated the high throughput discovery of SNPs [8-12]. Developing a large set of SNP markers for genome analyses in rainbow trout will facilitate fine mapping of QTL and will improve the identification and exploitation of genes affecting important traits and enable selective breeding through genomic selection.

Since 2002, the National Center for Cool and Cold Water Aquaculture (NCCCWA) has selectively bred rainbow trout broodstock for improvement of aquaculture production traits. The primary objectives have been to reduce the negative impacts of specifc bacterial pathogens on rainbow trout culture and to improve growth performance [13-15]. Currently, additional economically important traits such as stress tolerance [16,17] and meat/fillet quality are being evaluated. Molecular genetic technologies have the potential of increasing the rate of genetic gain of traditional selective breeding schemes [18]. Most production traits are controlled by multiple genes and inherited as quantitative traits. Analysis of quantitative trait loci (QTL) to identify loci for use in marker assisted selection (MAS) strategies can be used to optimize genetic improvement of desired traits [18]. To this end the NCCCWA is developing molecular resources for rainbow trout, including cDNA and BAC (Bacteria Artificial Chromosome) libraries, microsatellite markers and a linkage map [19-21]. The map was based on a panel of five families that represent the starting genetic material of the selective breeding program. At present, the linkage map consists of 1,124 microsatellite loci falling into 29 linkage groups, with an average sex distance of 2,927 cM and an average resolution of 2.6 cM. A recent linkage disequilibrium (LD) study by Rexroad *et al*. [22] found significant LD blocks at distances of 2 cM or less, implying that, to be used in QTL mapping, there is a need of a larger number of markers to be included in the map.

Expressed Sequence Tags (ESTs) have been used for SNP discovery in several non-model organisms, including salmonids [23-27]. However, when using EST data for SNP identification, the salmonid pseudo-tetraploid genome [28] can lead to assemblies of paralogous sequences and false positive SNP identification [24,26,27]. Initially, we attempted an SNP discovery approach based on alignments of ESTs. The Genbank UniGene Build #22 http://www.ncbi.nlm.nih.gov/sites/entrez?db=unigene presents over 200,000 Rainbow trout EST sequences. We first used custom bioinformatic pipelines to assemble the available ESTs and discover SNPs in rainbow trout. The occurrence of the genome duplication led to the assembly of paralogous sequences and resulted in a high number of false positive putative SNPs. Next, we sequenced genomic DNA based on EST sequences which proved to be a more efficient method for SNP discovery in rainbow trout, genomic DNA sequencing would allow the representation of the entire genome, and not just the transcriptomes, as that is the case with ESTs. However, this procedure was also extremely time consuming and not suitable for high throughput SNP discovery and it became clear that an alternative approach would be necessary to achieve the goal of a dense set of markers spanning the entire rainbow trout genome. In this study, we present the use of a high-throughput methodology to discover SNPs in rainbow trout based on pyrosequencing technology to generate data from a reduced representation library (RRL) constructed with genomic DNA pooled from 96 unrelated rainbow trout individuals, and evaluate the efficiency of this approach.

## Results

### Construction of Reduced Representation Libraries

**Figure 1:**
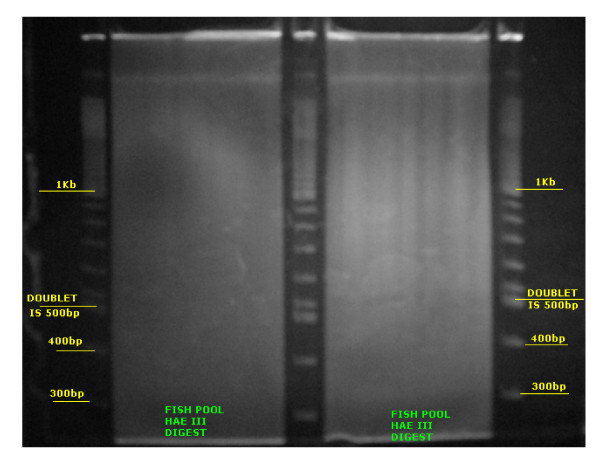
**Gel image of rainbow trout DNA digested with *Hae*III**. DNA fragments between 400 and 500 bp were excised from both lanes containing *Hae*III digested pooled DNA. Slices were cut every 2 mm or approximately 20 bp.

Restriction enzymes *Hpy*CH4V and *Bst*UI did not entirely digest the rainbow trout DNA, therefore, they were not considered for further procedures. Trout DNA of an individual animal was completely digested with *Alu*I, *Bst*UI and *Hae*III. In contrast with bovine DNA[9], and similar to swine[12], repetitive DNA bands were not observed when separating the fragmented DNA in polyacrylamide gels with any of the three enzymes. The restriction enzyme *Hae*III (Figure 1) was selected for the library construction based on analysis of sequence data of 960 clones containing fragments from the approximately 440 bp size range: 94% of the sequenced fragments were unique and only 12% of the unique sequences matched other sequences when blasted against the NCBI salmonids nucleotide DNA database, indicating acceptably low content of repetitive DNA in the fragment pool.

After digesting the pooled DNA from 96 unrelated individuals with *Hae*III, the 440 bp fragments were selected for library construction (3.2 μg of fragment DNA was recovered from the preparative gel). An aliquot of the recovered fragments was cloned and Sanger sequenced before proceeding with the library construction protocols: 89% of 1,269 sequenced fragments were unique and 33% of the analyzed bases masked to the cGRASP salmon repeat masker database http://lucy.ceh.uvic.ca/repeatmasker/cbr_repeatmasker.py.

### Sequencing results and SNP discovery

The Genome Sequencer FLX system produces about 400,000 reads (250 bp) per run, therefore, to cover 1% of the trout genome and to achieve a 6× average coverage, 4.5 runs were carried out, which resulted in 2,044,864 reads that contained a total of 459,825,391 bases for an average of 217 bp/read. The distribution of read lengths is rather asymmetric with a thick tail on the shorter side of the most common read length, which is 242 bp. After repeat masking, culling reads with less than 50 consecutive base pairs of non-repetitive content, and trimming repetitive ends, the result is 1,128,655 reads with an average length of 206 bp. Less than 15% of these reads contained internal repetitive content. The Newbler assembly of the 2 million unmasked reads resulted in 149,096 contigs with an average length of 259 bp and an average depth of coverage of 5.9. One-quarter of the contigs covered the length of the restriction fragments by connecting the reads from each end that overlap sufficiently in the middle. The rest of the contigs had lengths consistent with the read lengths. The estimate of the number of loci given in table 1 counts each long contig as one locus and each short contig as half a locus. Multiplying the estimated number of loci by the length of the restriction fragments indicates that 42.3 million base pairs of the trout genome were represented in this study. For reasons described below, a second assembly of the reads was constructed using the Velvet algorithm. The computational hardware available at the time did not allow an assembly of all the reads, but did enable an assembly of the smaller set of masked, culled and trimmed reads, resulting in 125,801 contigs with an average length of 192 bp/contig and an average depth of coverage of 6.4. Only 9% of the contigs spanned the restriction fragments. Using ssaha2 to compare the Velvet and Newbler assemblies showed that 77% of the Velvet contigs matched (minimum score of 80) to Newbler contigs.

**Table 1:** Sequence data analysis results

	Newbler1	Newbler2	Velvet
**No. of contigs**	149,096		125,081

**No. of loci**	94,000		69,000

**Read coverage**	5.9×		6.4×

**Putative SNPs**	47,128	22,022	33,624

**No. of contigs (SNPs)**	24,627	13,140	20,963

In order to discover SNPs present only in non-repetitive regions, the set of masked reads were mapped onto the two assemblies, with 75% mapping to the Newbler assembly and 63% mapping to the Velvet assembly. Both assemblies had similar distributions of coverage depth: ~40% less than 4×, ~10% greater than 10× and ~50% 4-10×. The average read depth at SNPs was skewed to the high end, ~9×. Despite the similarities, the two methods discussed thus far produced widely different predictions for the number of SNPs: 187,916 for Newbler and 52,942 for Velvet. The distribution of the SNPs across the contigs reveals that the vast majority of SNPs in the Newbler set came from contigs with 5 or more predicted SNPs. Filtering out such SNPs from both sets leaves 47,128 for Newbler and 33.624 for Velvet. Previous experience with SNP discovery in swine using a reduced representation library showed a much more reasonable distribution of putative SNPs across the assembled contigs [12]. One of the two attempts to lower the high rate of false positives was to use Velvet to create a different assembly. The other was to add an additional layering of filtering at the mapping stage (method N2 as described in the Methods section). N2 predicted 23,923 SNPs before filtering out the contigs with 5 or more SNPs and 22,022 after applying the filter. Results of the three analyses are summarized in Table 1.

A comparison of the SNPs from the three methods was done on a contig by contig basis, allowing for imperfect matches between velvet contigs and newbler contigs, as well as the occasionally varying numbers of predicted SNPs for the contigs. While compiling the lists of testable SNPs, some moderate filtering was performed to ensure sufficient quality and quantity of flanking sequence. As stated above, the two assemblies were more alike than different, yet the largest subgroup of SNPs is V1, indicating that the contigs unique to the Velvet assembly contain either a rich source of false positives or the best source of true SNPs. The next largest subgroup is N1, despite the fact that most of its potential SNPs were filtered out due to their presence in contigs with 5+ SNPs. The false positives seem to be infiltrating the unfiltered contigs as well. The third largest subgroup contains the SNPs predicted by all three methods, giving some assurance that the three approaches are similar enough to warrant comparison of validation rates as a means to incrementally improve the computational methods for SNP discovery. Even though the reads mapped in N2 were a subset (48%) of those mapped in N1, N2 contained SNPs not predicted in N1. These were from contigs that had 5 or more SNPs predicted by N1 but fewer than 5 predicted in N2. SNPs from each of the subgroups were tested and the validation results are discussed in the next section.

### SNP validation

GoldenGate genotyping assays resulted in the validation of 183 (48%) of the tested putative SNPs (384); 46 (12%) were homozygous; 4 (1%) showed paralogous sequences without allelic variation (all samples were heterozygous). It was not possible to obtain satisfactory genotyping results from the remaining sequences (39%), due to either unacceptable genotyping quality or the presence of paralogous sequences with allelic variation, therefore not conclusive to contemporary approach. Table 2 shows the validation rates within each group of predicted SNPs, which gives an indication of the merits of each computational method. The three highest validation rates all involved Velvet contigs, and three of the four lowest rates involved Newbler contigs that did not have a Velvet homologue, indicating that Velvet built a superior assembly with the difficult trout genome. Within the Newbler assembly that did not overlap the Velvet assembly, N2 produced better results than N1, but within the contigs common to both assemblies, N1 performed better than N2 (with the added condition that V1 co-predicted a SNP).

**Table 2:** Number of genotyped and validated markers from the three data analysis approaches.

Approach	Tested		Validated	
	
	#markers	%	#markers	%
**N1**	47	12.2	17	36

**N2**	46	12.0	22	48

**N1N2**	47	12.2	15	32

**V1**	100	26.1	53	53

**V1N1**	47	12.2	30	64

**V1N2**	47	12.2	18	38

**V1N1N2**	50	13.1	28	56

**Total**	**384**	**100**	**183**	**48**

N1, N2 and V1 represent SNPs predicted by only one of those three analyses; N1N2, V1N1, V1N2, and V1N1N2 indicate SNPs predicted by multiple analyses. Tested percentage refers to the number of markers in each group compared to all; the rate of validation within each approach is given as the validated percentage.

Call frequencies; minor allele frequencies and expected/observed heterozygosis of the validated SNPs are reported in Additional file 1. Call frequencies were higher than 90% for all markers and minor allele frequencies ranged from 1.3% to 49.7%. The double haploid samples were of great assistance for the validation process. Both Whale Rock samples were monomorphic for all validated markers; Skookumchuck, Hot Creek and Swanson were polymorphic for only one marker (OMS00033, OMS00163 and OMS00108, respectively) and Klamath for three markers (OMS00018, OMS00015 and OMS00108). The WGA DNA sample was successfully genotyped, 100% genotypes being equal to the original sample. Using SNaPshot, four SNPs were cross-validated. Genotyping results of the double-haploid fish were equal in both methods and 96% of the genotypes from the discovery samples were also equivalent.

BLAST analysis of the potential SNP sequences revealed that 1201 (1.5% of the total sequences) are associated with 790 ESTs/TCs in the rainbow trout transcriptome (>98% homology over at least 100 bp, E-value < 7.0E-41). The validated SNP sequences have 4 sequences associated with rainbow trout transcripts including proteins involved in lipocalin-type prostaglandin metabolism and immune system-Toll-like receptors (Table 3).

**Table 3:** Validated SNPs annotation.

OMS	GENE	Percentage_homology	E_VALUE	Annotation
OMS00035	CU065093	98.01	1E-101	AAI16452-B-cell CLL/lymphoma 9 [Homo sapiens]

OMS00028	TC158345	99.01	1E-104	CAG02508-unnamed protein product [Tetraodon nigroviridis]

OMS00080	TC139092	99.49	6E-106	Unknown

OMS00122	TC155403	99.49	3E-105	ACI66580-Lipocalin precursor [Salmo salar]

Validated markers were also genotyped using the NCCCWA mapping families and 167 markers were polymorphic and mapped to the rainbow trout second generation map[21]. In Additional file 2, the position of the SNP markers in the linkage map is indicated. The positions are based on the two-point distance of the SNP markers to the closest linked marker in the map with the highest confidence in map location. The newly developed markers were distributed along all linkage groups of the rainbow trout map; there are at least two markers in each linkage group.

## Discussion

In our first attempts to find SNPs in rainbow trout we evaluated multiple bioinformatic pipelines for their ability to detect SNPs from EST data (unpublished). The occurrence of the genome duplication resulted in many assemblies of paralogous sequences, which led to the identification of a large proportion of false positives (data not shown). In addition, a high number of the putative markers were not validated due to PCR amplification problems caused by incorrect assemblies of sequences; presence of introns in the amplicons and unspecific binding of primers (multiple loci). Similar results were observed in other salmonid species [23,26,27] and were attributed to the amplification of both paralogs of the same loci. A customized SNP discovery process using only 3' ESTs was then developed, which used stringent assembly parameters (98% identity; at least 50 bp overlap) were set to overcome issues associated with the genome duplication. 3' EST sequences were selected to avoid intron/exon boundaries; increase primer binding specificity and to raise the probability of finding SNPs in UTRs. However, the results were not improved and no markers were validated. The next approach was to sequence genomic DNA based on ESTs sequences, which proved to be an effective method of SNP identification in rainbow trout. Twenty putative SNPs were tested and 12 SNPs representing 10 different genes were validated on the double haploid samples; 5 of the validated SNPs were successfully genotyped on the 96 NCCCWA broodstock unrelated individuals (data not shown). However, this methodology is extremely time consuming and not suitable for high throughput SNP discovery.

Using a high throughput technology, over 20,000 putative SNPs were identified, 384 were tested and 183 validated; 90% of the validated markers were placed and widely distributed in the rainbow trout linkage map. A BLAST analysis was performed to identify coding regions in validated SNP sequences and 2% of the validated markers were associated with relevant genes. SNPs found within or near a coding sequence, are of particular interest because they are more likely to alter the biological function of a protein. This class of SNPs is especially important for species without a genome sequence such as aquaculture species. Gene-associated SNPs serve as suitable markers for mapping in comparative genome studies and in MAS of economically important traits [29,30].

Recent reports of using highly parallel sequencing methodologies in combination with reduced representation libraries (RRL) for SNP discovery in cattle and swine reported validation rates above 92% [9,12]. These species do not have the complication of recent whole genome duplication that is a prominent feature of the salmonid genome, which resulted in the dramatically reduced validation rate of approximately 48% reported here. Nevertheless, the RRL approach proved much more efficient than previous approaches used for trout SNP detection, providing tens of thousands of putative SNPs at a fraction of the cost of generating them by PCR and Sanger sequencing and in a much shorter time. Future work will attempt to revise the method to more efficiently avoid the class of putative SNPs resulting from comparisons between paralogous loci, by sequencing RRL produced from a double haploid strain in parallel to the pool of discovery, diploid animals. Additional gains may be made by refinement of filtering strategies by incorporating flanking sequence data more effectively, especially as developments in the pyrosequencing platform increase read lengths later this year permitting creation of RRL with longer fragments and increasing the ability to discriminate paralogs. We anticipate that future studies using RRL and pyrosequencing will approach the 90% success of other organisms, although we recognize that the tetraploid genome of rainbow trout will necessitate a much larger ratio of DNA sequenced to valid SNPs discovered than in species with less complex genomes.

## Conclusion

The use of reduced representation libraries and pyrosequencing technology proved to be an effective strategy for the discovery of a high number of SNPs in rainbow trout. Over 20,000 putative SNPs were discovered and 384 were tested, resulting in a 48% validation rate. A hundred and eighty three markers were validated and 167 (43.5%) of those markers were polymorphic and placed in the rainbow trout linkage map. According to the validation results, it could be implied that at least 10,000 putative SNPs from this data set would be validated and useful in future genomic studies. However, it is still necessary to dramatically decrease the false discovery rate.

## Methods

### Construction and sequencing of Reduced Representation Libraries

Five different restriction enzymes (*Alu*I; *Bst*UI; *Hae*III; *Hpy*CH4V and *Rsa*I) having four-base recognition target sequences and producing fragments with blunt ends were tested for the library construction. Digestions of 1 mg of DNA from a single fish were performed for each enzyme as suggested by the manufacturer (New England Bio Labs). The digested DNA was separated on 3 mm thick 5% nondenaturing polyacrylamide gels. DNA fragments between 400 and 500 bp were excised from the gels (slices were cut every 2 mm or approximately 20 bp), fragments were eluted using a modified [31] "crush and soak" procedure and DNA was purified by alcohol precipitation. For trial Sanger sequencing to identify the best enzyme for reduced representation library construction, the fragments were ligated into the pSTBlue-1 vector of the Perfectly Blunt Giga Cloning kit (Novagen). DNA sequencing was carried out with an ABI Prism 3100 Genetic Analyzer. The ability to completely digest the trout genomic DNA and the number of DNA repetitive elements present in the selected fragment size range were evaluated for each enzyme.

Approximately equal amounts of DNA from 96 unrelated individuals from the NCCCWA 2005 and 2006 brood years [22] was pooled prior digestion with *Hae*III. Complete digestions of 1 mg of DNA from the pool (3 U enzyme/μg) were performed overnight at 37°C. DNA fragments were separated and eluted from the gel following the procedures previously described. Excised fragments approximately 440 bp in length were selected to construct the library; 600 ng of DNA obtained from the preparative gel was used for construction of the library and ligated to the sequencing adapters provided in the GS FLX library construction kit (Roche Applied Science). From this point, the library was prepared and sequenced on the GS FLX as per the manufacturer's protocols.

### Sequence analysis and SNP discovery

Three independent SNP discovery approaches were used. The first two, which were called N1 and N2, were initiated by creating a reference sequence set by using the Newbler algorithm (version 1.1.03, provided with the GS FLX sequencer) to assemble all the unmasked sequence data into contigs. The individual reads were then screened for known repetitive elements and repeat masked using the cGRASP RepeatMasker database http://lucy.ceh.uvic.ca/repeatmasker/cbr_repeatmasker.py and sequences with at least 50 consecutive non-repetitive bases were mapped onto the assembly using ssaha2[32]. N2 differs from N1 in that the mappings for N2 were filtered so that only those with at least 90% sequence identity over at least 80% of the full length of the read were mapped onto the reference assembly. This step was done to decrease the likelihood of reads being mapped to duplicated regions of the genome. The third approach (V1) used the Velvet algorithm [33] for the assembly; in this case, all the sequence data was repeat-masked first, and the reads that contained at least 50 consecutive non-repetitive bases were used as input data. This same set of masked and filtered reads were then mapped back onto the Velvet assembly using ssaha2. The next step in all three approaches was to use ssaha_pileup [32] to detect sequence variation among the mapped reads. To be included as putative SNPs, both the major and the minor allele had to be detected at least twice each, without a third allele being observed. All SNPs from contigs that presented more than 5 putative SNPs (1 putative SNP/100 bp) were discarded, as a compromise balancing number of SNPs discovered and false discovery rate, since contigs with multiple apparent SNPs are more likely to represent paralogous loci (discarding loci with only two putative SNPs greatly decreases number of apparent SNPs but likely increases validation rate; allowing higher numbers adds additional SNPs but also increases false discover rate). Additionally, flanking sequence of at least 50 bp in one side (with a limit of 10% unknown bases) and 10 bp on the other side was required, in order to provide sufficient flanking sequence data to support assay design in the Illumina system to be used for genotyping.

### SNP Validation

Illumina GoldenGate assays [34] were used to evaluate a fraction of the discovered SNPs. A set of 384 putative SNPs was randomly chosen (from among those with the highest scoring flanking sequences) to represent each one of the three sequence analysis methods (N1: 47; N2: 46 and V1: 100) and putative SNPs found in more than one analysis were also tested (N1 and N2: 47; V1 and N1: 47; V1 and N2: 47; V1, N1 and N2: 50).

Genomic DNA from 85 of the discovery panel fish and 98 samples from five NCCCWA mapping families [21] were used for the validation assays. In addition, seven double haploid samples from the Arlee, Hot creek, Klamath, Whale rock female, Whale rock male, Skookumchuck and Swanson clonal lines [35,36] were genotyped and used as a control to eliminate the paralogous sequences that can erroneously be identified as SNPs. In some genotyping projects, the amount of DNA available for the assays is limited, and it is necessary to use Whole-Genome Amplified (WGA) DNA, as has already been used in GoldenGate assays [37]. A protocol based on the method described by [38] was used to prepare one WGA sample, which was added to the SNP validation panel, its respective template was also genotyped for comparison.

The BeadStudio software version 3.2 (Illumina) was used to analyze the Goldengate assays SNP data. Genotyping data quality was assessed following the guidelines suggested by Illumina [39]. The no-call threshold used was 0.25 and all the samples used for validation had at least a 0.85 Call rate score. A cross-validation assay was done using the ABI SNapshot method; four SNPs were randomly selected and the 96 discovery samples and the seven double-haploid individuals were genotyped.

A local BLAST search using the sequences of the validated SNPs against the rainbow trout transcriptome data from TIGR (BioEdit software at default settings http://www.mbio.ncsu.edu/BioEdit/BioEdit.html) was performed to identify coding regions.

### Linkage analysis

Segregating data from the NCCCWA mapping families was used to anchor the polymorphic validated markers into the second generation rainbow trout linkage map [21]. Genotype data combined for both sexes were formatted using MAKEPED of the LINKAGE [40] program and checked for inconsistencies with Mendelian inheritance using PEDCHECK [41]. RECODE [42] and LNKTOCRI [43] were used to assemble the data into CRIMAP [44] format. SNP genotype data were added to that of Rexroad et al [21] and MULTIMAP [45] was used to conduct two-point linkage analyses to identify the closest markers from the published map which were ordered at the highest level of significance.

## Authors' contributions

CCS contributed to project design, was responsible for construction of the reduced representation library and Illumina genotyping for SNP validation, and drafted the manuscript. TS shared in conception and design of the project, assisted with library preparation, and led the sequencing and data pipeline aspects. RW was responsible for project design relating to SNP identification, performed the sequence assembly and developed the list of putative SNP. RV performed the linkage analysis. MS annotated the SNP sequences. JY contributed to overall project design. CR shared in conception and design of the project, led linkage analysis, coordinated the project and shared responsibility for manuscript preparation. All authors reviewed the manuscript.

## Supplementary Material

Additional file 1 Additional Table S1. Validated SNPs information. Click here for file

Additional file 2 Additional Table S2. Mapped SNPs. Click here for file
